# Streamlined, automated protocols for the production of milligram quantities of untagged recombinant human cyclophilin-A (hCypA) and untagged human proliferating cell nuclear antigen (hPCNA) using ÄKTAxpress™

**DOI:** 10.1016/j.pep.2009.12.001

**Published:** 2010-05

**Authors:** Cornelia Ludwig, Martin A. Wear, Malcolm D. Walkinshaw

**Affiliations:** The Edinburgh Protein Production Facility (PPF), Centre for Translational and Chemical Biology (CTCB), Wellcome Centre for Cell Biology (WCCB), University of Edinburgh, Michael Swann Building, King’s Buildings, Mayfield Road, Edinburgh EH9 3JR, UK

**Keywords:** Cyclophilin-A, PCNA, Automated protein purification, Liquid chromatography, ÄKTAXpress™

## Abstract

We developed streamlined, automated purification protocols for the production of milligram quantities of untagged recombinant human cyclophilin-A (hCypA) and untagged human proliferating cell nuclear antigen (hPCNA) from *Escherichia coli*, using the ÄKTAxpress™ chromatography system. The automated 2-step (cation exchange and size exclusion) purification protocol for untagged hCypA results in final purity and yields of ⩾93% and ∼5 mg L^−1^ of original cell culture, respectively, in under 12 h, including all primary sample processing and column equilibration steps. The novel automated 4-step (anion exchange, desalt, heparin-affinity and size exclusion, in linear sequence) purification protocol for untagged hPCNA results in final purity and yields of ⩾87% and ∼4 mg L^−1^ of original cell culture, respectively, in under 24 h, including all primary sample processing and column equilibration steps. This saves in excess of four full working days when compared to the traditional protocol, producing protein with similar final yield, purity and activity. Furthermore, it limits a time-dependent protein aggregation, a problem with the traditional protocol that results in a loss of final yield. Both automated protocols were developed to use generic commercially available pre-packed columns and automatically prepared minimal buffers, designed to eliminate user and system variations, maximize run reproducibility, standardize yield and purity between batches, increase throughput and reduce user input to a minimum. Both protocols represent robust generic methods for the automated production of untagged hCypA and hPCNA.

## Introduction

The demand placed on protein production strategies, in terms of the amount and purity of the protein products produced and the need to develop reliable and robust generic purification protocols, has increased massively in the post genome-sequencing era [1]. This is especially so for the increasing pace and scope of structural genomics and drug discovery programs, where there is very often a need to generate tens of milligrams of highly pure protein on a regular basis, with as little batch variation as possible [1–5]. Modern liquid chromatography instruments have become increasingly automated [6–8] and associated separation media increasingly sophisticated [9,10], resulting in the development of effective methodologies for preparative protein purification, with increased throughput, becoming more and more routine [1]. However, more often than not, these protocols are developed as bespoke methods for a particular protein or family of proteins, and make use of some very specific differences in biophysical properties particular to the individual protein(s). Although extremely effective, these bespoke methods do not translate particularly well into generic or high-throughput purification strategies. In addition to this, the vast majority of purification strategies involve more than one step (even for protocols utilizing some form of affinity enrichment as the first step [11]) and most lab-scale chromatography instrumentation normally only handles a single chromatographic step at a time. As such, multistep purification protocols frequently involve time-consuming manual processing, including, for example, SDS–PAGE analysis of chromatograms and the subsequent pooling of the appropriate fractions, desalting or dialysis, concentration and application of the partially processed sample to the next chromatography column/step.

The ÄKTAxpress™ liquid chromatography system (GE Healthcare) was the first widely available lab-scale system designed to specifically help address these demands, allowing preparative milligram-level protein purification protocols of up to 4-steps to be automated and parallelized [6]. A peak detection algorithm and a set of internal capillary loops allow peak fractions from intermediate steps to be collected automatically and processed through subsequent chromatography steps in the protocol. The system is able to process protein samples through a variety of affinity, ion-exchange, desalt and gel-filtration chromatography applications, using a set of generic pre-packed column types, with the option to include an on-column affinity tag removal cleavage step, as part of the protocol [6]. One major advantage of this system is the non-modular design (apart from the columns and an external loop for the application of a protease, no other components are removed or added), which means each instrument is essentially operationally identical. All instruments have the same flow path delay volumes, the same gradient delay volumes, the same set of pre-defined column types and the same sets of limited user-editable protocol design parameters. This, in theory, means that there is no appreciable difference in way the instruments are run between laboratories, and translation of an already developed purification protocol from one instrument to another will not require any further system/user specific optimization that often plagues researchers trying to replicate a method using a different chromatography system to that used in a published protocol.

The vast majority of protocols using the ÄKTAxpress™ utilize affinity chromatography as the first step [6,12], as this enrichment technique is easily scaled to fit the loop volume and sample handling restrictions of the instrument (the standard configuration has five 10 ml loops with a nominal 7.5 ml collection and process volume) for subsequent desalt or gel-filtration steps in the protocol. However, the presence of an affinity tag and optimizing its subsequent removal (very often a requirement for structural work) sometimes causes as many problems as the development of the purification protocol itself (even when utilizing the very widely exploited poly-histidine tag [11]). The small size and highly charged/polar nature of poly-histidine tags usually ensures that tagged-proteins structure and activity is rarely affected, but there are instances of this not being the case [13,14]. Two proteins of particular interest to us as potential therapeutic targets – human cyclophilin-A (hCypA)^1^ and human proliferating cell nuclear antigen (hPCNA) – have exhibited problems as tagged-proteins.

Cyclophilins, found in all prokaryotes and eukaryotes and in all cellular compartments, are involved in protein assembly and cellular signaling [15,16]. Their cellular function requires their peptidyl-prolyl isomerase (PPIase) activity that accelerates protein folding/unfolding [17]. Most cyclophilins also bind the immunosuppressive drug cyclosporin-A (CsA) [18]. In humans, the resulting hetero-dimeric complex inhibits calcineurin, blocking the signal transduction pathway to interleukin production [19,20]. Human cyclophilins have also recently become interesting drug targets in a number of diseases including HIV [21,22] and HCV infection [23–27], malaria [28,29], ischemia [30–32], immunosuppression, as well as showing potent anti-nematode effects [16,33,34].

PNCA, an ubiquitous eukaryotic protein, is essential for DNA replication, DNA repair and plays major roles in the post-replicative processing of DNA [35,36]. Functional PCNA is a toroidal trimer capable of encircling double-stranded DNA, enhancing polymerase processivity by tethering the polymerase complex to the target DNA [37,38]. Many of the proteins that interact directly with PCNA are involved in the mechanics of DNA replication and repair [37,38]. PCNA also interacts with proteins involved in post-replicative processing and with cell cycle regulatory proteins such as Gadd45 and p21 (WAF1/Cip1) [35,39,40]. As a result of its central role in DNA replication and repair, PCNA has been highlighted [41] as a potential therapeutic target in a multitude of proliferative cancers.

Our laboratory has previously found that his-tags have adversely affected both the structure and function of cyclophilins and PCNA in a variety of activity and structural assays, as well as in a number of small molecule screening assays. In addition, tag cleavage has proved less than straightforward, for reasons undefined, and many of our small molecule screening assays and structural analyses require production of milligram amounts of very pure and active protein on a regular basis. As a direct result, we developed reliable and robust automated purification protocols, for the production of milligram amounts of very pure untagged recombinant human CypA, by easily adapting an existing protocol [42], and for untagged human PCNA, by development of a novel 4-step protocol, using the ÄKTAxpress™ liquid chromatography system. Both automated protocols use generic commercially available pre-packed columns and automatically prepared minimal buffers, essentially eliminating user and system variations. They also maximize the run reproducibility and standardize the yield and purity between batches, increase throughput and reduce user input to a minimum. The automated 4-step protocol for hPCNA saves 4 working days over the traditional method, greatly increasing the overall productivity of the protocol. These two protocols further highlight the versatility of the ÄKTAxpress™ liquid chromatography as a way of standardizing lab-scale/process protein production.

## Materials and methods

### Materials

All chemicals used were of the highest grade available commercially.

### Plasmid construction

Expression plasmids for recombinant hCypA (*pSW3-001*) and hPCNA (*pT7-PCNA*) were constructed as described [42] and [43], respectively. *pT7-PCNA* was kindly provided by Dr. Emma Warbrick (University of Dundee, UK).

### Protein expression and purification

All purification was performed on ÄKTAxpress™ and ÄKTA-Purifier 100 UPC (GE Healthcare) equipment at 6 °C. The ÄKTAxpress™ instruments were used in a standard configuration with 10 ml collection loops. HiPrep 26/60 S-200 HR gel-filtration and HiPrep 26/20 Desalt columns (GE Healthcare) were attached to the system with the recommended lengths of 1.0 mm i.d. Tefzel^®^ tubing. All buffers, with the exception of *Buffer-H*, were generated on an ÄKTA-Purifier 100 system fitted with an on-line *Buffer Prep* function, a 2 ml mixing chamber, using a flow rate of 30 ml min^−1^ and the standard buffer recipes (Table 1) supplied with the instrument software (UNICORN v 5.11, GE Healthcare). Proteins were detected by absorbance at 280 nm. Culture volumes used were 1L in all cases.

### Traditional purification of hCypA

The following protocol was adapted from the method described by Wear et al. [42] to run with the appropriate pre-packed column types for the ÄKTAxpress™ instrument and a minimal buffer system. Recombinant hCypA was expressed and purified to homogeneity from OverExpress C41 BL21(DE3) *Escherichia coli* (Lucigen) transformed with the *pSW3-001* plasmid, grown shaking (260 rpm) at 37 °C for 16 h in Overnight Express Instant TB Medium (Novagen) containing carbenicillin (100 μg ml^−1^). Cells were harvested by centrifugation and resuspended at 10% (w/v) in ice-cold *Buffer-A* (Table 1), plus protease inhibitors. Lysis was performed at 6 °C by a single passage through a Constant Systems Cell Disruptor TS Series Benchtop instrument set to 22 kPSI. Cellular debris was removed by centrifugation at 50,000*g* for 1 h at 4 °C, and the filtered (0.22 μm) supernatant was then applied to a 5 ml HiTrap SP HP column (GE Healthcare) pre-equilibrated in *Buffer-A*, at 4 ml min^−1^*.* The resin was washed with 10 column volumes of *Buffer-A* at a flow rate of 4 ml min^−1^ and bound proteins were eluted with a gradient from 0% to 40% *Buffer-B* over 10 column volumes (Table 1) at 4 ml min^−1^, collecting 2 ml fractions. Relevant fractions (hCypA elutes between 13% and 22% *Buffer-B*) were analysed using SDS–PAGE (4–20% acrylamide), pooled (between 14% and 22% *Buffer-B*) and the 10 ml sample flushed from a 10 ml loop with one loop volume plus an additional 3 ml onto a HiPrep 26/60 S-200 HR gel-filtration column (*V*_t_ ∼ 320 ml) (GE Healthcare) pre-equilibrated in *Buffer-C* (Table 1). The column was run at 1.6 ml min^−1^ and fraction collection was set to start after 0.6 column volumes and continue for a further 0.2 column volumes, collecting 2 ml fractions. Fractions containing hCypA (hCypA peak *V*_e_ = 223.2 ml) were pooled, concentrated to ∼500 μM and stored on ice. hCypA was ⩾93% pure as judged by densitometric analysis of SDS–polyacrylamide gels.

### Automated purification of hCypA using ÄKTAXpress™

Recombinant hCypA was expressed and lysed as described above. *Buffer-A*, *Buffer-B* and *Buffer-C* (Table 1) and default system settings for a 2-step ion-exchange (IEX), gel-filtration (GF) protocol, in the cold, were used unless otherwise stated. Following lysis, the clarified supernatant was applied to an ÄKTAXpress™ system fitted with a 5 ml HiTrap SP HP column and a HiPrep 26/60 S-200 HR gel-filtration column. The IEX column was eluted with a gradient from 0% to 40% *Buffer-B* over 10 column volumes. During elution of the IEX column, collection was set to percentage, collecting between 15% and 21% *Buffer-B* into a single loop. The peak detection parameters were left as default for level and slope for the GF step, run in *Buffer-C* at 1.6 ml min^−1^, and fraction collection was set to start after 0.6 column volumes and continue for a further 0.2 column volumes, collecting 2 ml fractions. Invariably, fractions A7–B1 of the hCypA peak were pooled, concentrated to ∼500 μM and stored on ice. hCypA was ⩾93% pure as judged by densitometric analysis of SDS–polyacrylamide gels.

### Traditional purification of hPCNA

OverExpress C43 BL21(DE3) *E. coli* (Lucigen) transformed with the *pT7-PCNA* plasmid were grown shaking (250 rpm) at 37 °C in LB media containing carbenicillin (100 μg ml^−1^) until the *A*_600_ was ∼0.7 and over-expression of recombinant hPCNA was induced by addition of IPTG to 1 mM and growth for a further 3 h at 37 °C. Cells were harvested by centrifugation and resuspended at 10% (w/v) in ice-cold *Buffer-D* (Table 1) plus protease inhibitors. Lysis was performed at 6 °C by a single passage through a Constant Systems Cell Disruptor TS Series Benchtop instrument (Constant Systems) set to 25 kPSI. Cellular debris was removed by centrifugation at 50,000*g* for 1 h at 4 °C, and the supernatant applied to a 5 ml HiTrap Q HP column (GE Healthcare) pre-equilibrated in *Buffer-D*, at 5 ml min^−1^. Following loading, the column was washed with 15 column volumes 27% *Buffer-E* (Table 1), followed by a 2.7 column volume gradient from 27% to 54% *Buffer-E* and then a 5 column volume gradient from 54% to 57% *Buffer-E*, at 5 ml min^−1^. Eluted protein was collected continuously in 1 ml fractions and analysed using SDS–PAGE (4–20% acrylamide). Relevant fractions were pooled from 50.7% to 55.5% *Buffer-E*. The protein pool (∼17 ml) was split into two equal aliquots and loaded onto a HiPrep 26/10 Desalting column (GE Healthcare) pre-equilibrated in *Buffer-F* (Table 1), run at 8 ml min^−1^ and collecting 1 ml fractions. Relevant fractions containing protein were pooled and loaded onto a 5 ml HiTrap Heparin HP column (GE Healthcare), pre-equilibrated in *Buffer-F*, at 5 ml min^−1^. Following loading, the resin was washed with 5 column volumes of 18% *Buffer-G* (Table 1), followed by an 8.2 column volume gradient from 18% to 100% *Buffer-G*, at 5 ml min^−1^ collecting 2 ml fractions from 35.8% to 65.4% *Buffer-G*. Eluted protein was analyzed by SDS–PAGE (4–20% acrylamide) and relevant fractions pooled. The resulting 15 ml protein pool was then split into two equal 7.5 ml aliquots and loaded (7.5 ml sample flushed from a 10 ml loop with one loop volume plus an additional 3 ml) onto a HiPrep 26/60 Sephacryl S-200 HR gel-filtration column pre-equilibrated with *Buffer-H* (Table 1), at 1.6 ml min^−1^. Relevant fractions containing hPCNA were analyzed by SDS–PAGE, pooled from 110 to 128 ml, concentrated to 100 μM and stored on ice. hPCNA was ⩾93% pure as judged by densitometric analysis of SDS–polyacrylamide gels.

### Automated purification of hPCNA using ÄKTAXpress™

Recombinant hPCNA was expressed and lysed as described above. *Buffer-D*, *Buffer-E*, *Buffer-F*, *Buffer-G* and *Buffer-H* (Table 1) and default system settings for a 4-step ion-exchange (IEX), desalt (DS), affinity (AF), gel-filtration (GF) protocol, in the cold, were used unless otherwise stated. Following lysis, the clarified supernatant was applied to an ÄKTAXpress™ system fitted with a 5 ml HiTrap Q HP column, a HiPrep 26/10 Desalting column, a 5 ml HiTrap Heparin HP column and a HiPrep 26/60 S-200 HR gel-filtration column. Following loading, the 20 column volume *wash-out-unbound-material* parameter was set to 0 and the column was washed with 15 column volumes 27% *Buffer-E*, followed by a 2.7 column volume gradient from 27% to 54% *Buffer-E* and then a 5 column volume gradient from 54% to 57% *Buffer-E*, at 5 ml min^−1^. During elution of the IEX column, collection was set to percentage, starting at 50.6% *Buffer-E* collecting 10 ml into a single loop. During elution of the DS column with *Buffer-F*, peak detection parameters were set to start at 80 mAU, with a slope factor of 10 mAU min^−1^, a peak max of 0.15 and peak end of 50 mAU, with collection into two 10 ml loops. The contents of both loops were loaded onto the AF column with a *peak-injection-flush-volume* of 20 ml *Buffer-F*. Following loading, the AF column was washed with 5 column volumes of 18% *Buffer-G*, followed by an 8.2 column volume gradient from 18% to 100% *Buffer-G*. During elution of the AF column with *Buffer-G*, collection was set to percentage, starting at 39.7% *Buffer-G* collecting 10 ml into a single loop. The contents of the AF loop were loaded onto the GF column with a *peak-injection-flush-volume* of 13 ml. During elution, peak detection parameters were left as default for level and slope, with collection set to start after 0.26 column volumes and continue for a further 0.21 CV, collecting 2 and 1 ml fractions. Invariably, fractions A7–C11 of the hPCNA peak were pooled, concentrated to ∼100 μM and stored on ice. hPCNA was ⩾87% pure as judged by densitometric analysis of SDS–polyacrylamide gels.

### Peptidyl-prolyl isomerase (PPIase) assay

This colorimetric assay, performed essentially as described [44,45], determines the rate of the *cis* to *trans* conversion of the peptidyl-prolyl amide bond in the substrate *N*-succinyl-Ala-Ala-Pro-Phe-*p*-nitroanilide (sAAPF-pNA). Selective enzymatic hydrolysis of sAA*trans*PF-pNA by α-chymotrypsin [46] releases *p*-ntitroaniline (pNA), the accumulation of which is monitored by the absorbance at 400 nm. sAAPF-pNA, dissolved in 470 mM LiCl in 2,2,2-trifluroethanol (TFE) at 200 mM, was diluted to 4 mM in LiCl/TFE immediately before use. Reactions were conducted at 6 °C in 50 mM HEPES, pH 8.0, 100 mM NaCl; 5 mM DTT, in a total reaction volume of 1 ml, essentially as described [44,45]. Final concentrations of hCypA and AAPF-pNA were 15 and 100 μM, respectively. The initial linear portion of the slopes (0–1.2 s) were converted to rates in μM s^−1^ using the absorbance at 400 nm and the extinction coefficient *ε*_400 nm_ = 10,050 M^−1^cm^−1^. The IC_50_ was determined by a least squares fit of Eq. (1) to plots of the initial reaction rate (background thermal isomerisation rate subtracted), *V*_o_ (in μM s^−1^), *versus* the concentration of CsA.

(1) $$V_{\text{o}}=V_{\infty }+(V_{\text{zero}}-V_{\infty })/\{({1+([\text{CsA}]/{\text{IC}}_{50})}^{\mathrm{Slope}})\}$$

*V*_∞_ is the reaction rate at infinite inhibitor concentration (fixed at 0), *V*_zero_ is the initial velocity in the absence of inhibitor, [CsA] is the concentration of CsA, IC_50_ is the concentration of CypA that causes 50% inhibition and *Slope* is the Hill number. Correction for substrate competition was performed using Eq. (2).

(2) $$K_{\text{ds}}={\text{IC}}_{50}/1+([\text{AAPF}]/K_{\text{m}})$$

[AAPF] is the initial AA*cis*PF-pNA concentration (mean = 51.6 μM) and *K*_m_ is the substrate Michaelis–Menten constant (798 ± 69 μM).

### Miscellaneous

SDS–PAGE was performed as described [47]. The molecular weights of hCypA, hPCNA and CsA are 18,012, 28,769 and 1202.12 Da, respectively. Protein concentration was determined by measurement of absorbance at 280 nm and calculated using the extinction coefficient 8490 M^−1^ cm^−1^, for hCypA and 16,305 M^−1^ cm^−1^, for hPCNA, or by BCA protein assay (Pierce) with bovine serum albumin (BSA) as a standard.

## Results and discussion

### hCypA

**Fig. 1:**
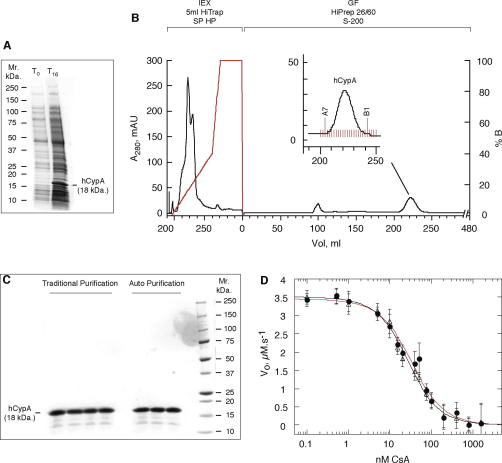
Automated purification of hCypA. (A) SDS–polyacrylamide gel (4–20% gradient) illustrating the typical levels of soluble hCypA over-expressed from OverExpress C41 BL21(DE3) *E. coli* grown for 16 h at 37 °C in Overnight Express Instant TB Medium. hCypA makes up ∼6% of the total soluble protein. *T*_o_, soluble cell extract at mid log phase immediately after inoculation (*A*_600 nm_ ≈ 0.5); *T*_16_, soluble cell extract following 16 h of growth shaking (260 rpm) at 37 °C. (B) Typical chromatogram for the automated 2-step purification of hCypA using ÄKTAXpress™. The pre-packed columns used are illustrated above the corresponding section of the chromatogram; IEX – ion-exchange, GF – gel-filtration. Solid black; *A*_280 nm_ in mAU (left axis). Solid red; NaCl gradient in % *Buffer-B* (right axis). The inset details the region of the gel-filtration column elution from which fractions were collected. Indicated fractions A7–B1 were pooled. (C) SDS–polyacrylamide gel (4–20% gradient) illustrating the final purity levels of hCypA purified by both traditional and automated protocols. Both methods produce protein of ⩾93% purity (determined by gel densitometry). Five μg total protein was loaded in each lane. Four independent traditional runs and 3 independent automatic runs are shown, illustrating the excellent reproducibility of both methods and the excellent comparable purity between the methods. Molecular weight markers are shown to the right of the gel. (D) Inhibition of the PPIase activity (*V*_o_ in μM^−1^ s^−1^) of hCypA (15 nM) by cyclosporin (CsA). hCypA purified by either the traditional or automatic method shows the same high specific activity. Open triangles, black line, automatically purified hCypA; solid circles, red line, traditionally purified hCypA. The solid lines are a best fit to Eq. (1) (see Materials and methods). The values for the equilibrium dissociation constant for cyclosporin inhibition (*K*_i_) are 24.3 ± 4.2 nM, for traditionally purified hCypA and, 19.7 ± 2.8 nM, for automatically purified hCypA, agreeing very well with literature values [42,44,48]. (For interpretation of the references to color in this figure legend, the reader is referred to the web version of this paper.)

Although the 2-step protocol (cation exchange and gel-filtration) for the purification of untagged recombinant human CypA (see Fig. 1A for typical soluble expression levels of hCypA) described by Wear et al. [42] generates very pure and active protein, there is still significant manual intervention involved in processing the samples through to purity. We streamlined the original [42] traditional protocol (see Materials and methods for details) by introducing several significant improvements. This was achieved primarily by using generic pre-packed columns, a set of automatically generated minimal buffers (*Buffer-A*, *Buffer-B* and *Buffer-C*; Table 1), containing only buffer salt and NaCl, eliminating the need for any additional components (except for the addition of a protease inhibitor cocktail, added to the cell suspension mix just prior to high-pressure lysis), and a significantly increased flow rate compared to the previous method (4 and 1 ml min^−1^, respectively) [42]. We switched the cation exchange column of the first step to a generic commercially available column type (a 5 ml HiTrap SP HP column; GE Healthcare), with a greatly reduced bed volume (5 ml compared to the previous ∼50 ml). This, coupled with the increased flow rate, reduced the run time to less than 15% of the previous protocol (∼40 min compared to ∼300 min) [42]. The loss of bed volume had no adverse effect on the amount of sample we typically needed to process and the elution peak of hCypA was, in general, between 7 and 8 ml in volume. Cell extract from up to 3 l of original *E. coli* culture could be similarly processed without any apparent loss of purity or saturation of the dynamic binding capacity of the column (∼40–60 mg protein ml^−1^ of wet resin).hCypA invariably eluted between 13% and 22% *Buffer-B* and, following SDS–PAGE analysis, fractions collected between 14% and 22% *Buffer-B* were pooled and processed over a HiPrep 26/60 S-200 HR gel-filtration column (GE Healthcare), loading with a 10 ml loop with an additional 3 ml flush volume and run in *Buffer-C* (Table 1). hCypA invariably eluted as a symmetrical peak with an elution volume of 223.2 ± 0.3 ml (mean ± SD, *n* = 4). The streamlined traditional methodology was extremely reproducible; Fig. 1C illustrates the final protein obtained from 4 independent repeat runs. It routinely produced hCypA with a final purity of ⩾93% and final yield of ∼5 mg per litre of original bacterial culture (Table 2, Fig. 1C). The yields and purity we obtained with this method were similar to those obtained using the previous method [42]. However, the batch purity and yield variability was significantly reduced and the total processing time (from cell pellet to pure protein) was reduced by at least half.

We next took the streamlined traditional protocol and translated it onto an ÄKTAXpress™ system fitted with a 5 ml HiTrap SP HP column and an HiPrep 26/60 S-200 HR gel-filtration column, with only a few minor changes to the run parameters previously optimized for the traditional methodology. Recombinant hCypA was expressed (Fig. 1A) and lysed identically to the traditional method. *Buffer-A*, *Buffer-B* and *Buffer-C* (generated automatically; Table 1) and default system settings for a 2-step ion-exchange, gel-filtration protocol, in the cold, were used except for the following changes. The ion-exchange column was eluted with a gradient from 0% to 40% *Buffer-B* over 10 column volumes, with collection set to percentage, collecting between 15% and 21% *Buffer-B* into a single loop. This sample was then processed onto the gel-filtration column run in *Buffer-C*. Fraction collection was set to start after 0.6 column volumes and continue for a further 0.2 column volumes, collecting 2 ml fractions. Fig. 1B illustrates the typical chromatogram obtained from such an automated run. The inset shows the detail of the hCypA peak; the elution volume for hCypA was 222.9 ± 0.12 ml (mean ± SD, *n* = 3), essentially identical to the traditional method, and the fractions finally pooled from these runs were invariably A7–B1 (Fig. 1B). Analysis of the final pool of protein indicated hCypA was ⩾93% pure (Fig. 1C), with independent repeat runs of the automatic method producing protein with essentially identical purity and yield (Fig. 1C, Table 2), illustrating the excellent reproducibility of the automated protocol. Furthermore, the specific activity of purified hCypA is very high; Fig. 1D shows a comparison of the PPIase activity of protein purified either by the traditional method or the automated methods. The values for the equilibrium dissociation constant for CsA inhibition (*K*_i_) are 24.3 ± 4.2 nM, for traditionally purified hCypA, and 19.7 ± 2.8 nM, for automatically purified hCypA, agreeing very well with literature values [42,44,48].

Due to the ability to pool more judiciously and manually process the sample between columns with the traditional method, the final yields of hCypA generated were typically ∼5–7% higher than the automated method; yields were ∼5 mg per litre of original culture (Table 2). However, the automated 2-step purification protocol involves very much less user intervention and the processing time from cell pellet to final pure protein is less than 12 h, including all primary sample processing and column equilibration steps. This saves almost half a working day of user intervention over the traditional method making the automatic method far more efficient. Despite this small loss of yield compared to the traditional protocol, our automated purification protocol for untagged recombinant hCypA using the ÄKTAXpress™ system represents a very rapid, robust, reproducible and, most importantly, an improved generic methodology for the production of milligram quantities of very pure protein.

### hPCNA

**Fig. 2:**
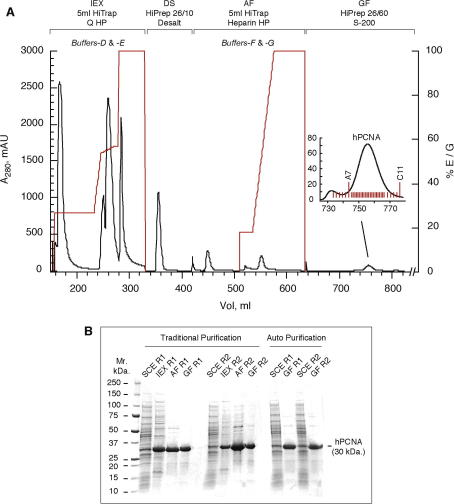
Automated Purification of hPCNA. (A) Typical chromatogram for the automated 4-step purification of hPCNA using ÄKTAXpress™. The pre-packed columns used are illustrated above the corresponding section of the chromatogram; IEX – ion-exchange, DS – desalt, AF – affinity, GF – gel-filtration. Solid black; *A*_280 nm_ in mAU (left axis). Solid red; elution gradient in % *Buffer-E* or *Buffer-G* (right axis), IEX and AF step, respectively. The buffer pairs used are indicated above the appropriate portion of the chromatogram. The inset details the region of the gel-filtration column elution from which fractions were collected. Indicated fractions A7–C11 were pooled. (B) SDS–polyacrylamide gel (4–20% gradient) illustrating the final purity levels of hPCNA purified by both manual (⩾93%) and automated (⩾87%) protocols (determined by gel densitometry). The final purity from 2 independent traditional and 2 independent automatic runs are shown, illustrating the excellent reproducibility of both methods. Five μg total protein was loaded in each lane. SCE, soluble cell extract; R1, run 1; R2, run 2. Molecular weight markers are shown to the right of the gel. (For interpretation of the references to color in this figure legend, the reader is referred to the web version of this paper.)

Novel protocols for the purification of recombinant untagged hPCNA from *E. coli* were developed from scratch (see Materials and Methods for full details). We first developed a traditional 4-step ion-exchange (IEX), desalt (DS), affinity (AF), gel-filtration (GF) methodology, again utilizing generic pre-packed columns (suitable for use on the ÄKTAXpress™ system), and a set of automatically generated minimal buffers (*Buffer-D*, *Buffer-E*, *Buffer-F*, *Buffer-G*, Table 1; Fig. 2). The IEX column elution uses a relatively complex 4-step gradient profile: a wash immediately following sample application with 15 column volumes of 27% *Buffer-E*, followed by a 2.7 column volume gradient from 27% to 54% *Buffer-E*, followed by a 5 column volume gradient from 54% to 57% *Buffer-E*, followed by a step to 100% *Buffer-E*. Relevant hPCNA fractions from 50.7% to 55.5% *Buffer-E* were pooled and further processed. This gradient profile proved critical for ensuring hPCNA eluted with as narrow an elution peak as possible (between 16 and 17 ml), while at the same time limiting the level and number of contaminants, allowing an easy 2-repeat run processing through the subsequent desalting step without further concentration. hPCNA purified by this method was ⩾93% pure as judged by densitometric analysis of SDS–polyacrylamide gels (Fig. 2B) and typically yielded ∼2.5 mg per litre of original bacterial culture (Table 2). Despite the fact that very pure protein could be reproducibly obtained (Fig. 2B), the traditional method requires a very considerable amount of manual intervention and typically takes 5 full working days to process the protein from cell pellet through to purity. Furthermore, a significant amount of protein (∼1–2 mg) was lost over the course of purification due to a time-dependent aggregation and resulting precipitation problems. These effects were only partially alleviated by the addition of glycerol to the chromatography buffers. Thus, in an attempt to reduce the loss of protein, to greatly reduce the amount of user input to a minimum and to further standardize the purification, we streamlined this method further by translating it into a fully automated protocol on the ÄKTAXpress™ system.

Clarified *E. coli* cell lysate containing recombinant hPCNA was applied to an ÄKTAXpress™ system fitted with a 5 ml HiTrap Q HP column (GE Healthcare), a HiPrep 26/10 Desalting column (GE Healthcare), a 5 ml HiTrap Heparin HP column (GE Healthcare) and a HiPrep 26/60 S-200 HR gel-filtration column (GE Healthcare). *Buffer-D*, *Buffer-E*, *Buffer-F*, *Buffer-G* (generated automatically; Table 1) and *Buffer-H* (Table 1), and default system settings for a 4-step ion-exchange, desalting, affinity, gel-filtration protocol, in the cold, were used except for the following changes (see Materials and methods for full details). Following loading, column was washed with 15 column volumes 27% *Buffer-E* (setting the default *wash-out-unbound-material* parameter to 0), followed by a 2.7 column volume gradient from 27% to 54% *Buffer-E* and then a 5 column volume gradient from 54% to 57% *Buffer-E*, followed by a step to 100% *Buffer-E* at 5 ml min^−1^ (Fig. 2A). During elution of the IEX column, collection was set to percentage, starting at 50.6% *Buffer-E*, collecting 10 ml (instead of the default 7.5 ml) into a single loop; the protein was further processed through a desalting step. During elution of the DS column with *Buffer-F*, peak detection parameters were set to start at 80 mAU, with a slope factor of 10 mAU min^−1^, a peak max of 0.15 and peak end of 50 mAU, with collection into two 10 ml loops. The contents of both loops were loaded onto a 5 ml Heparin AF column with a *peak-injection-flush-volume* of 20 ml *Buffer-F*. Following loading, the AF column was washed with 5 column volumes of 18% Buffer-*G*, followed by elution with an 8.2 column volume gradient from 18% to 100% *Buffer-G*. During elution of the AF column with *Buffer-G* collection was set to percentage, starting at 39.7% *Buffer-G*, collecting 10 ml, again into a single loop. We found the minor loss of sample arising from using the entire loop volume, due to laminar flow where velocity increases as the center of the tube is approached, was more than compensated by the increased yield that resulted from collecting 10 ml of sample through the core of the eluted protein peak for both the IEX and AF columns. The contents of the entire AF loop were then loaded onto the GF column with the default *peak-injection-flush-volume* of 13 ml. During elution peak detection, parameters were left as default for level and slope with collection set to start after 0.26 column volumes and continue for a further for 0.21 CV, collecting 2 and 1 ml fractions (Fig. 2A). Fractions, invariably A7–C11, of the hPCNA peak were pooled, concentrated to ∼100 μM and stored on ice. hPCNA was ⩾87% pure as judged by densitometric analysis of SDS–polyacrylamide gels (Fig. 2B, Table 2).

Independent repeat runs of the fully automated method produced protein with the same degree of high purity (Fig. 2B), illustrating the excellent reproducibility of the automated protocol. Streamlining and translation of the purification protocol onto an ÄKTAXpress™ system reduces the processing time to a fraction of that taken by the traditional method. The automated run takes less than 24 h, including all primary processing and column equilibration steps, with minimal user input to process the sample through from cell pellet to final purity, compared to the 5 full working days of the traditional methodology. This is particularly important; hPCNA is prone to time-dependent aggregation and the automated protocol limits the loss of protein to this effect. As a direct result, the final yield of protein from a litre of original culture obtained from the automated method was typically ∼40% greater than the traditional method (3.6 mg *versus* 2.5 mg; Fig. 2B, Table 2), purely as a result of being able to process and purify the protein very much more quickly. Our automated purification protocol for untagged recombinant hPCNA using the ÄKTAXpress™ system represents a novel generic methodology for the production of milligram quantities of pure protein.

### Concluding remarks

We have developed reliable and robust automated purification protocols, for the production of milligram amounts of very pure untagged recombinant human CypA, by easily adapting an existing protocol [42], and for untagged human PCNA, by development of a novel 4-step protocol, using the ÄKTAxpress™ liquid chromatography system. Both automated protocols use generic commercially available pre-packed columns and automatically prepared minimal buffers (essentially eliminating user error and system variations), helping to further maximize run reproducibility and standardize the yield and purity between batches. They also increase throughput and reduce user input to a minimum; the automated 4-step protocol for hPCNA saves 4 working days over the traditional method increasing the overall productivity of the protocol. These two protocols also further highlight the versatility and high degree of reproducibility of the ÄKTAxpress™ liquid chromatography system as a lab-scale production system.

## Figures and Tables

**Table 1:** List of buffers, composition and recipes used for chromatography steps.

Buffer	Buffer composition	Buffer stock solutions^a^
*Buffer-A*	24 mM HEPES, pH 6.8	Line A1 – 100 mM HEPES
Line A2 – 100 mM NaOH
Line B1 – ddH_2_O
Line B2 – 2 M NaCl
*Buffer-B*	24 mM HEPES; 1 M NaCl, pH 6.8	Line A1 – 100 mM HEPES
Line A2 – 100 mM NaOH
Line B1 – ddH_2_O
Line B2 – 2 M NaCl
*Buffer-C*	7.8 mM Na_2_HPO_4_; 150 mM NaCl, pH 7.5;	Line A1 – 30 mM Na_2_HPO_4_
Line A2 – 100 mM HCl
Line B1 – ddH_2_O
Line B2 – 2 M NaCl
*Buffer-D*	15.5 mM 1-methyl-piperazine; 15.5 mM Bis–Tris; 7.8 mM Tris, pH 8.5	Line A1 – 50 mM 1-methyl-piperazine; 50 mM Bis–Tris; 25 mM Tris
Line A2 – 100 mM HCl
Line B1 – ddH_2_O
Line B2 – 2 M NaCl
*Buffer-E*	15.5 mM 1-methyl-piperazine; 15.5 mM Bis–Tris; 7.8 mM Tris; 1 M NaCl, pH 8.5	Line A1 – 50 mM 1-methyl-piperazine; 50 mM Bis–Tris; 25 mM Tris
Line A2 – 100 mM HCl
Line B1 – ddH_2_O
Line B2 – 2 M NaCl
*Buffer-F*	5.1 mM Na_2_HPO_4_; 5.1 mM Formate Na; 10.2 mM Acetate Na, pH 5.5	Line A1 – 30 mM Na_2_HPO_4_; 30 mM Formate Na; 60 mM Acetate Na
Line A2 – 100 mM HCl
Line B1 – ddH_2_O
Line B2 – 2 M NaCl
*Buffer-G*	5.1 mM Na_2_HPO_4_; 5.1 mM Formate Na; 10.2 mM Acetate Na; 1 M NaCl, pH 5.5	Line A1 – 30 mM Na_2_HPO_4_; 30 mM Formate Na; 60 mM Acetate Na
Line A2 – 100 mM HCl
Line B1 – ddH_2_O
Line B2 – 2 M NaCl
*Buffer-H*	25 mM Tris; 25 mM NaCl; 0.5 mM EDTA, 10% glycerol, pH 7.5	Manually prepared

a Buffer recipe stock solutions and the ratio mixtures for the final buffer composition were generated as described in the UNICORN (v5.11, GE Healthcare) operating software.

**Table 2:** Purification of hPCNA and hCypA. Fractionation was performed on cell pellets obtained from 1 l of *E. coli* culture.

Fraction	Total protein (mg)^a^	Purity (%)^b^
*hCypA – Traditional*		
Supernatant	498	6
Pooled SP HP fractions	5.8	88
Pooled S-200 fractions	5.2	⩾93

*hCypA – Automated*		
Pooled S-200 fractions.	4.9	⩾93

*hPCNA – Traditional*		
Supernatant	630	11
Pooled Q HP fractions	71.4	32
Pooled Heparin HP fractions	12.7	89
Pooled S-200 fractions	2.5	⩾93

*hPCNA – Automated*		
Pooled S-200 fractions	3.6	⩾87

a Mean values from at least 2 individual repeat runs of the corresponding protocol. Protein concentration in the supernatant and the pooled fractions after each chromatographic step was determined by BCA protein assay, apart from the final S-200 pool where protein concentration was determined by *A*_280_ measurements.

b Determined by densitometry of appropriate lanes on reducing SDS–polyacrylamide gels (4–20% gradient).

## Footnotes

1 *Abbreviations used:* hCypA, human cyclophilin-A; hPCNA, human proliferating cell nuclear antigen; PPIase, peptidyl-prolyl isomerase; CsA, cyclosporin-A; IEX, ion-exchange; GF, gel-filtration.
